# Application of the rapid prototyping technique to design a customized temporomandibular joint used to treat temporomandibular ankylosis

**DOI:** 10.4103/0970-0358.53031

**Published:** 2009

**Authors:** Suresh M. Chaware, Vaibhav Bagaria, Abhay Kuthe

**Affiliations:** Central India Institute of Medical Sciences, Bajaj Nagar, Nagpur, India; 1 Department of CAD/CAM, VNIT, Nagpur, India

**Keywords:** Ankylosis, CAD, Rapid prototyping, Temporomandibular joint, Total joint replacement

## Abstract

Anthropometric variations in humans make it difficult to replace a temporomandibular joint (TMJ), successfully using a standard “one-size-fits-all” prosthesis. The case report presents a unique concept of total TMJ replacement with customized and modified TMJ prosthesis, which is cost-effective and provides the best fit for the patient. The process involved in designing and modifications over the existing prosthesis are also described. A 12-year- old female who presented for treatment of left unilateral TMJ ankylosis underwent the surgery for total TMJ replacement. A three-dimensional computed tomography (CT) scan suggested features of bony ankylosis of left TMJ. CT images were converted to a sterolithographic model using CAD software and a rapid prototyping machine. A process of rapid manufacturing was then used to manufacture the customized prosthesis. Postoperative recovery was uneventful, with an improvement in mouth opening of 3.5 cm and painless jaw movements. Three years postsurgery, the patient is pain-free, has a mouth opening of about 4.0 cm and enjoys a normal diet. The postoperative radiographs concur with the excellent clinical results. The use of CAD/CAM technique to design the custom-made prosthesis, using orthopaedically proven structural materials, significantly improves the predictability and success rates of TMJ replacement surgery.

## INTRODUCTION

Total joint replacement surgery for hip and knee has proven to be the most successful surgical procedure, promising better function and less pain than before for people suffering from chronic pain or dysfunction due to injury, illness or even genetics. However, over the past few years, success was mixed when it came to total replacement of the jaw joint – technically, the temporomandibular joint (TMJ). Because of the many differences in the structure and shape of the human skull, it is difficult to replace a jaw joint successfully with anything other than a highly customized prosthesis (artificial joint). By contrast, prostheses for knees and hips are fairly similar and vary little, except for the size of the patient. We present a case report of total TMJ replacement with customized and modified TMJ prosthesis, which is cost-effective and a perfect fit for the patient. The process involved in designing and modifications over the existing prosthesis are also described.

## MATERIAL AND METHODS

### Case report

**Figure 1(a-f):**
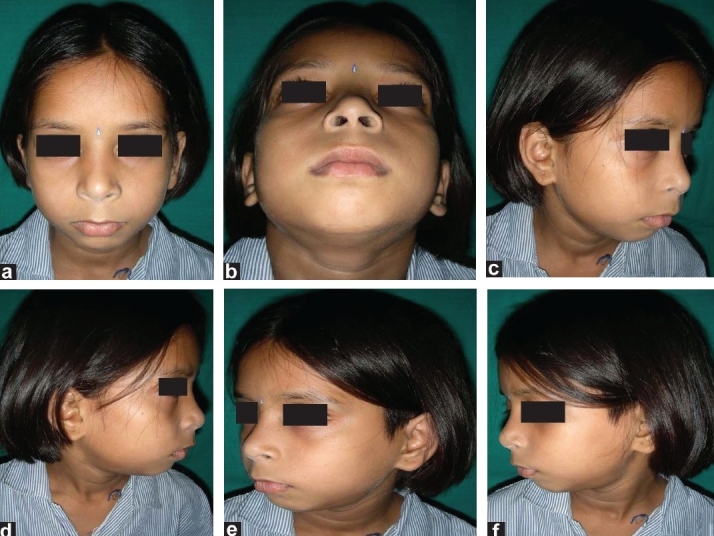
Preoperative

A 12-year-old female presented for treatment of left unilateral TMJ ankylosis [Figure 1a–f]. The ankylosis was caused secondary to trauma sustained during her early childhood.

**Figure 2:**
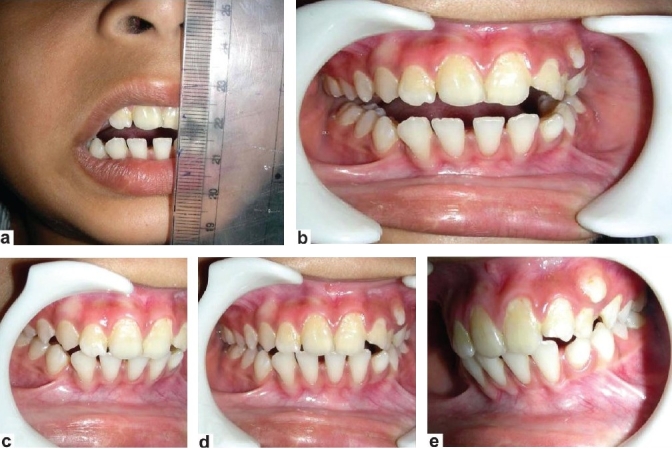
(a-b) Preoperative mouth opening was about 3 mm; (c-e) Shows occlusion was about class I (preoperative)

The patient was disabled due to difficulty in speaking coherently, impaired mastication and poor oral hygiene. Clinically, her interincisal opening distance was only 3 mm. Radiographs suggested a bony adhesion on the left side of the TMJ. A three-dimensional (3D) computed tomography (CT) scan confirmed the 10/27/2009features of bony ankylosis of left TMJ with minimal changes in right TMJ. There was no significant facial deformity. Occlusion was of class I. A decision to replace the left TMJ with customized TMJ prosthesis was taken in view of her problems [Figures 2a–e].

### Designing and customizations of TMJ prosthesis

**Figure 3:**
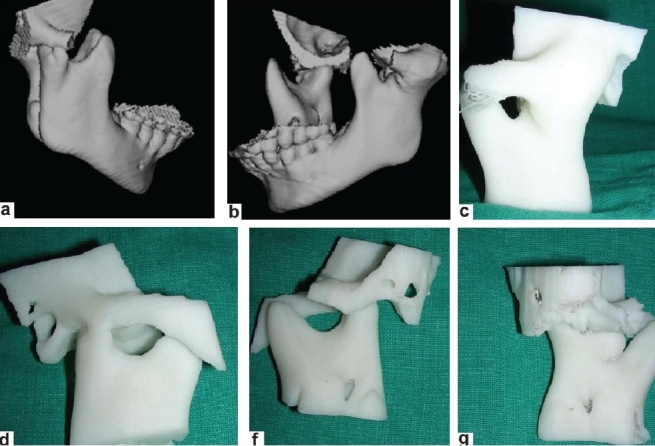
(a-b) Three-dimensional computed tomography scan of the patient; (c-g): From these computed tomography images, an SLE model is made by the rapid prototypic technique

**Figure 4:**
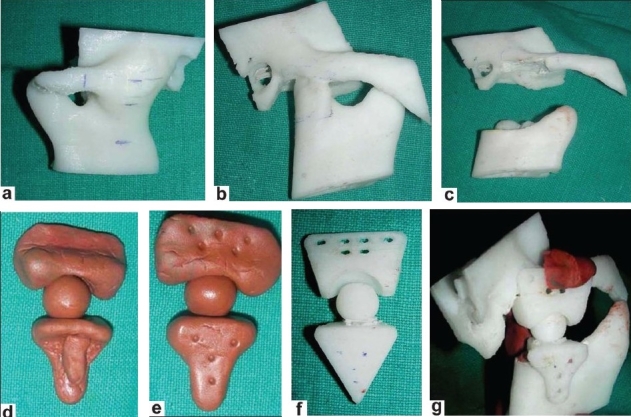
(a-b) Marking of gap arthoplasty is performed; (c) Gap arthoplasty is performed on the model; (d-e) Clay model of prosthesis is made; (e) This clay model is digitalized to make an SLE model of prosthesis; (f) SLE model of prosthesis; (g) Accuracy of prosthesis confirmed on the model

**Figure 5:**
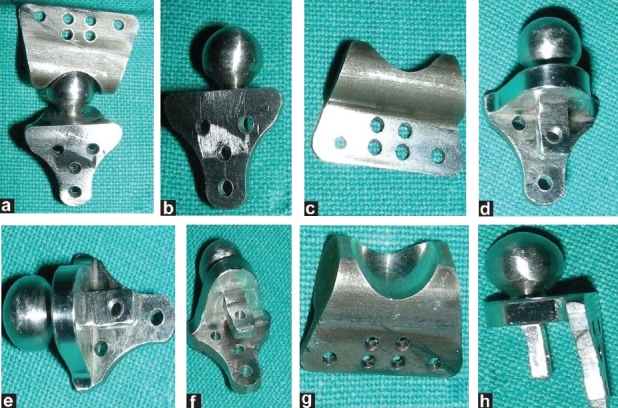
(a-b) Marking of gap arthoplasty is performed; (c) Gap arthoplasty is performed on the model; (d-e) Clay model of prosthesis is made; (f) This clay model is digitalized to make an SLE model of prosthesis; (g) SLE model of prosthesis; (h) Accuracy of prosthesis confirmed on the model

Creating an accurate TMJ prosthesis was carried out in the following different steps [Figure 3a–f, 4a–g and 5a–h]:

Initially, a preoperative CT scan of the jaws and jaw joints was obtained using a standard protocol of 2 mm thickness slices. Using the CT data, a 3D plastic model of the TMJ and associated jaw structures was made using the stereolithographic technology. The mandible was spatially repositioned on the model to correct the functional and aesthetic misalignment problems. From these models, an accurate measurement of the distance of gap arthroplasty was performed.

The condyles were removed and the necessary bony recontouring of the fossa and mandibular ramus was carried out on the plastic ABS model to rehearse the procedure and also to plan the customization preoperatively. A custom-made total joint prosthesis conforming to the patient's specific anatomical morphology and jaw interrelationships is then fabricated on clay. This clay model is digitalized using CAD–CAM Software (Materialise, Belgium) and transferred to the Rapid Prototyping machine to make an accurate SLE model of prosthesis. On confirming accuracy of prosthesis, actual prosthesis is fabricated.

### Surgery

**Figure 6:**
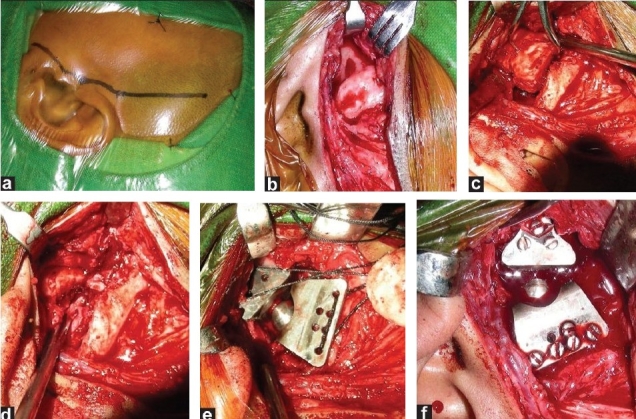
(a-f) Surgery–replacement; (a) Preauricular incision; (b) Ankylosed temporomandibular joint exposed; (c) Gap arthoplasty is being performed; (d) Gap arthoplasty completed; (e) Prosthesis introduced in defect; (f) Final fitting of prosthesis

The TMJ and mandibular ramus was approached via a left preauricular incision. Condylectomy, debridement and bone recontouring were accomplished as previously determined on the steriolithographic (SLE) model. Intermaxillary fixation (wiring of the upper and lower jaws together) was then performed. The fossa component of the prosthesis was inserted through the preauricular incision and stabilized to the zygomatic arch with three to four 2 mm-diameter screws. The mandibular component was also inserted via the same incision and fixated to the lateral surface of the ramus with four 2 mm-diameter screws. Interlocking was performed with a 2 mm screw [Figure 6a–f]. At completion of surgery, the intermaxillary fixation was removed to facilitate active jaw functions.

### Postoperative

**Figure 7(a-d):**
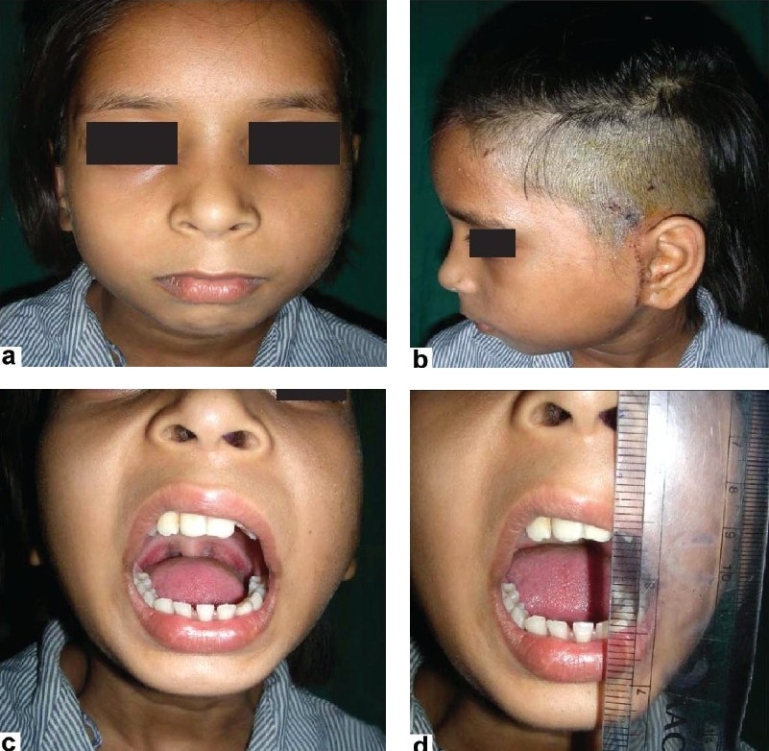
Postoperative–immediate, shows mouth opening of about 3 cm

**Figure 8(a-g):**
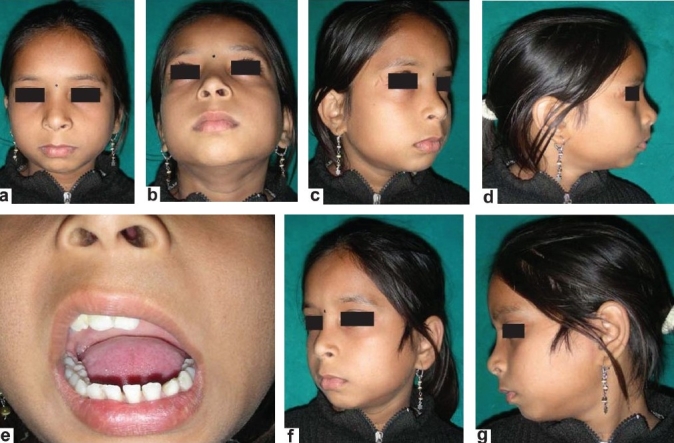
Postoperatve result after 3 years shows that mouth opening is about 3 cm with no facial deformity

Stitches were removed on the 5^th^ day and the patient was discharged on the 6^th^ day. Soft diet was allowed on the fifth postoperative day till 6 weeks. After 6 weeks, she was allowed normal diet avoiding very hard food. Mouth opening exercises were advised [Figure 7a–d and 8a–g].

## RESULTS

**Figure 9:**
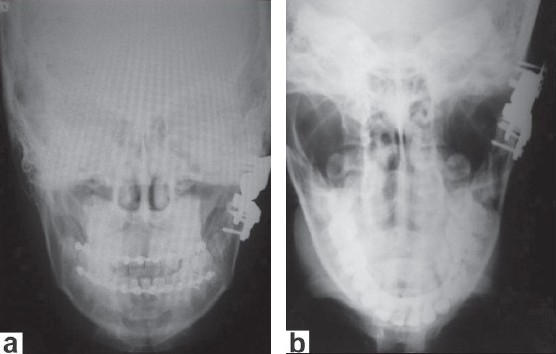
(a) Four-day postoperative X-ray shows that prosthesis is well fit; (b) Three-year postoperative X-ray shows that prosthesis is well in place and there are no signs of erosion at the skull base

Postoperative recovery was uneventful. Immediate postoperative mouth opening was 2.5 cm. Jaw movements were totally painless. After 3 years of surgery, she is pain-free, having mouth opening of about 3.5 cm and enjoying normal diet. Radiographs performed at 6 weeks, 3 months and 2 years showed a normal functioning joint without any loosening or migration. There was no evidence of instability [Figure 7a–d, 8a–g and 9a and b]. The postoperative scar has healed well and there is no evidence of any infection.

## DISCUSSION

TMJ is similar to a gynglymoarthrodial joint with an additional sliding type of movement. Technically, the ball portion is the mandibular condyle (jaw) and the socket portion is the fossa. There is a disc between the two bone segments, which allows the condyle to slide smoothly during a range of motion or while opening the mouth. Muscles keep the joint together and provide the force required to move the jaw. Mobility is accomplished by an initial hinge motion (straight vertical opening) as well as gliding forward (increasing vertical opening) and side to side.[1] Vertical movement is about 50 mm, with a 10 mm protrusion and 10 mm lateral movement.[2] The TMJ can deliver in chewing forces 7–23 Kgf for cheese and as high as 35 Kgf for peanuts. [3] These features make the joint unique and it is important to understand the anatomy, physiology and mechanics of the joint to improve the surgical techniques and design implants that will withstand various stress factors.

In 1963, Robert Christensen[4] first described a molded vitallium (cobalt–chromium) prosthesis that covered the glenoid fossa. This was secured to the skull with two to three screws and the meniscus was allowed to remain *in situ*, which he felt helped to prevent adhesions and lubricated and lessened joint trauma. At times the disc was unsalvageable and was removed. This process was also used with a condylar component. Christensen devised about 50 different sizes from cadaver skulls for a custom-fit in individual patients. Statistics showed a favourable outcome.

Morgan,[5,6] in 1971, first described a vitallium articular eminence implant of an acrylic condylar head and vitallium shank being utilized to replace an absent condyle. His total TMJ replacement was reported in 1976.

The only total joint prostheses that were still commercially available were the Christensen and Morgan devices. Although these devices were not food and drugs association (FDA) approved, they were still available to practitioners as “grandfathered” devices because they fell into the FDA preamendment of 1976.

One more type of total TMJ prosthesis that merits mention is the use of a polyoxymethylene (Delrin) condylar head that was affixed to a titanium mesh shank.[7]

Indications for TMJ surgery consist of internal derangement, degenerative joint disease, condylar fractures, neoplastic diseases, growth deformities, fibrous/bony ankylosis, rheumatoid or nonrheumatoid arthritis and hypomobility disorders.[8] Various factors can cause TMJ ankylosis, including trauma, systemic and local infections and neoplasms in the area. A higher incidence of posttraumatic ankylosis in children was reported by Laskin.[9]

In 1909, metatarsal bone was used in the first condylar reconstruction and the first costochondral graft was utilized in 1920.[10] The use of costochondral junction grafts from actively growing samples was recommended by Sarat and Robinson in 1956.[11] Long-term treatment with steroids may reduce the physical strength of the rib and there is risk of further fibrosis and even ankylosis.[12,13] In addition, the rib graft often sits lateral to the ramus and can be difficult to locate precisely in the glenoid fossa. For these reasons, alloplastic materials have been developed for total prosthetic replacement of the TMJ.

Henry and Wolford[14] reported a study on 107 patients where autogenous tissues were used to reconstruct the TMJ when previous Proplast–Teflon had been placed. This study also showed a significant increase in failure rates for all autogenous tissue groups as the number of prior TMJ surgeries increased. The use of a custom-made total joint prosthesis may improve the results in many of these conditions.

Reconstruction of the TMJ disc has also been carried out with dermis, auricular cartilage, freeze-dried dura and temporalis muscle and fascia. A temporalis muscle flap was first described in 1898 by Golovine for reconstruction of TMJ.[15] Autogenous bony tissues other than costochondral grafts that have been used were metatarsal, iliac crest, fibula, tibia, cranial bone and sternoclavicular grafts.

The only FDA-approved device for total joint TMJ reconstruction is the TMJ concepts total joint prosthesis. The use of this custom-made prosthesis, made with orthopaedically proven structural materials in combination with autogenous periimplant fat grafting, has significantly improved the predictability and success rates of treatment for the rehabilitation of complex TMJ patients.[16]

The CAD/CAM technique is a boon for a reconstructive surgeon. With the help of the CAD/CAM technique, actual life size models of mandible with skull were prepared on which mock surgery was carried out. With CAD/CAM, a perfect-fitted prosthesis was created.

The most custom fit of all joint prosthesis was the Techmedica CAD/CAM[17,18] custom computer-assisted design/computer-assisted make-up (Camarillo, CA, USA). Data indicate that the CAD/CAM Patient Fitted Total Temporomandibular Joint Reconstruction System has proved to be a safe and effective long-term management modality in the patient population surveyed for this study.

For a TMJ total joint prosthesis to be successful, the following structural and functional characteristics should be met:

1. biocompatible and functionally compatible materials,
2. low wear, flow and fatigue coefficients of articulating materials,
3. close adaptability to anatomic structures and function,
4. rigidly stabilized components,
5. corrosion-resistant, nonfragmenting and nontoxic materials,
6. low incidence of hypersensitivity,
7. posterior stop in the fossa component and
8. close tolerance of the screw and prosthesis hole diameter.[19]

A prosthesis that meets these criteria is extremely important in the long-term successful outcome of the reconstructive process.

### Characteristics of our prosthesis

1. The TMJ total joint prosthesis has two basic components: A fossa component and a mandibular component.
2. Ball and socket type of prosthesis.
3. The foss part is very stable and hence there are lesser chances of dislocation. Very stable joint.
4. Size of prosthesis is smaller.
5. Fixation area is less; hence, surgery is performed through the same incision.
6. For the first time, principle of INTERLOCKING is used in this prosthesis. Fixation is more solid and less surface area is required for fixation.
7. It is cost effective. Total cost of prosthesis is about INR 2500. (The cost for rapid prototyping and study model being about INR 5000.10,000).
8. It is a universal prosthesis. Can be used on either side.
9. The surgical time was diminished owing to perfect fit and same incision.
10. Fewer scars over the face.

Our prosthesis is a truly ball and socket type, having a ball mounted on the shoulder of the prosthesis. The socket is deep and accommodates it nicely. The prosthesis like Christensen, W. Lorenz, has a peg-like condylar end and the socket portion is open laterally. Hence, our prosthesis is more stable. In the commercially available prostheses, fixation depends on fixation screws making them larger in size and requiring more screws for fixation. The shoulder of our prosthesis sits on the cut end of the ramus. Hence, the whole stress is on the shoulder and ramus and not on the screw of fixation. This design makes it smaller in size commercially available prosthesis.

The principle of “Interlocking” was used making the fixation more firm. Though other prosthesis require two incisions, our customized prosthesis can be fitted through a single incision, thereby considerably reducing operative time. Our results show that our prosthesis is stable and can be a long-term solution for diseases of TMJ requiring total replacement.

The prosthesis as described has several advantages, but certain cautions and long-term studies are required before the prosthesis is put into widespread commercial application. One apparent drawback is the time taken for manufacturing the prosthesis, which could be up to 10 days; but given the elective nature of surgeries, in most cases, this does not pose a problem. Another potential cause of concern could be defining the indications for its uses, although the case described here demonstrates a good result at 2 years with use of the prosthesis in growing children. There needs to be a long-term assessment on a continuous basis for the functionality while using it in skeletally immature patients. The cost of prosthesis is expected to be around INR 2500, which seems to be akin to the pricing other available options.

## CONCLUSION

This being the first case, the patient is under regular follow-up. Short- term outcomes seem very promising. However, the long-term results are awaited.
